# Relationship between size of pharyngeal and palatine tonsils and apnea–hypopnea index in pediatric obstructive sleep apnea

**DOI:** 10.20407/fmj.2023-011

**Published:** 2024-02-15

**Authors:** Masamichi Kaneko, Masatoshi Hirata, Ayami Kimura, Hiroya Inada, Kazuki Shikano, Satoshi Ito, Takayuki Okano, Hiroshi Yatsuya, Seiichi Nakata

**Affiliations:** 1 Department of Otolaryngology and Sleep Medicine, Fujita Health University, School of Medicine, Nagoya, Aichi, Japan; 2 Department of Clinical Laboratory, Fujita Health University Bantane Hospital, Nagoya, Aichi, Japan; 3 Department of Public Health and Health Systems, Nagoya University Graduate School of Medicine, Nagoya, Aichi, Japan

**Keywords:** Obstructive sleep apnea, Apnea–hypopnea index, Risk factors for severe OSA, Pharyngeal and palatine tonsils, Combination

## Abstract

**Objective:**

To determine whether the combination of the pharyngeal tonsil grade and palatine tonsil grade results in differences in the apnea–hypopnea index (AHI) and to determine whether each parameter separately (pharyngeal tonsil grade and palatine tonsil grade) results in differences in severe obstructive sleep apnea (OSA).

**Methods:**

This cross-sectional study involved 107 children (mean age, 7.2 years; range, 4–12 years) suspected of having OSA because of snoring or sleep-related complaints. The patients underwent polysomnography, and their palatine and pharyngeal tonsils were graded.

**Results:**

In examining whether the palatine tonsils and pharyngeal tonsils could be risk factors for severe OSA, the adjusted odds ratios were 4.42 for palatine tonsil grade 4 versus 1–3 and 10.40 for pharyngeal tonsil grade 4 versus 1–3; both were highly statistically significant. We also found that the AHI when both the pharyngeal and palatine tonsils were grade 4 was higher than the AHI expected for the pharyngeal and palatine tonsils alone.

**Conclusions:**

The combination of grade 4 pharyngeal tonsils and grade 4 palatine tonsils resulted in an AHI much higher than the AHI of other combinations (pharyngeal tonsils grades 1–3 and 4, palatine tonsils grades 1–3 and 4). We believe that grade 4 pharyngeal tonsils and grade 4 palatine tonsils have a great influence on severe OSA and that grade 4 pharyngeal tonsils increase the AHI.

## Introduction

Obstructive sleep apnea (OSA) has been recognized as an important clinical entity in children.^1^ OSA is characterized by narrowing of the pharyngeal airway, resulting in repeated episodes of airflow cessation, oxygen desaturation, and sleep disruption.^2^ Enlargement of the pharyngeal tonsils and palatine tonsils frequently narrows the nasopharynx and oropharynx, respectively, leading to partial or total obstruction of the upper airways. The most common cause of OSA in children is pharyngeal and palatine tonsil hypertrophy.^3^ Palatine tonsil hypertrophy and pharyngeal tonsil hypertrophy are independent risk factors for OSA.^4^ However, not every child with large palatine and pharyngeal tonsils has symptoms of OSA. In addition, most studies have focused on the size of the pharyngeal and palatine tonsils; few studies have examined the combination of the pharyngeal and palatine tonsil grades.

Therefore, the main objectives of the present study were (1) to determine whether the combination of the pharyngeal tonsil grade and palatine tonsil grade results in differences in the apnea–hypopnea index (AHI) and (2) to determine whether each parameter separately (pharyngeal tonsil grade and palatine tonsil grade) results in differences in severe OSA.

## Methods

### Ethical considerations

This study was conducted in accordance with the Declaration of Helsinki and approved by the Ethics Review Committee of Fujita Health University (approval number: HM23-036). The patients’ parents were shown pictures of the equipment and devices involved in the polysomnography (PSG) examinations. We explained the possibility of releasing data for medical research and obtained written consent from the parents of all patients. If the child was unable to sleep, hypnotic medication was used to facilitate the test. Triclofos sodium syrup was used at a standard dosage of 20 to 80 mg/kg in accordance with the package insert.

### Patients

This cross-sectional study involved 107 children (mean age, 7.2 years; range, 4–12 years) suspected of having OSA because of snoring or sleep-related complaints who visited Bantane Hospital, Fujita Health University during the 44-month period between March 2014 and October 2017.

Initially, 122 children were diagnosed with OSA based on an interview and the results of PSG according to the criteria of the International Classification of Sleep Disorders III.^5^ Fifteen of the 122 children were excluded because of Down syndrome (n=1), Pierre Robin syndrome (n=2), lack of palatine tonsil measurement data (n=2) lack of pharyngeal tonsil measurement data (n=7), lack of both palatine and pharyngeal tonsil measurement data (n=2), and significant septal deformity (n=1). The remaining 107 children were enrolled in this study.

There are no uniform criteria for the severity of pediatric OSA based on the AHI obtained by PSG, nor are any criteria mentioned in current guidelines. Referring to several previous studies,^6–8^ we classified the severity of pediatric OSA into three levels: mild (AHI of 1–5), moderate (AHI of 5–10), and severe (AHI of ≥10).

### PSG

We conducted a comprehensive sleep study in a controlled environment with minimal background noise. The study involved standard PSG with various physiological measurements, including electroencephalogram parameters (F4-M1, C4-M1, and O2-M1) of the reference electrode derivation principle, electrooculogram, chin/anterior tibial electromyograms, nasal/mouth-breathing airflow analysis by thermistor and pressure sensors, chest wall/abdominal motion analysis using a strain gauge, snoring sound analysis using a microphone, oxygen saturation analysis by pulse oximetry, and electrocardiography.

The respiratory events were scored according to the American Academy of Sleep Medicine criteria for scoring various events during PSG.^9^ Using an oronasal thermal sensor, we identified obstructive apnea when there was a drop in the peak signal excursion by ≥90% of the pre-event baseline, lasting for at least two breaths during baseline breathing and associated with respiratory effort throughout the entire period of absent airflow. Using nasal pressure, we identified a respiratory event as hypopnea if the peak signal excursion dropped by ≥30% of the pre-event baseline, lasting for at least two breaths and associated with ≥3% desaturation from the pre-event baseline, or if the event was associated with an arousal.

### Assessment of pharyngeal and palatine tonsils

The most frequently used classification of enlarged palatine tonsils in children is Brodsky’s classification.^10^ Friedman’s classification^11^ of palatine tonsil hypertrophy originates from the success or failure of uvulopalatopharyngoplasty in adults. However, Mengi et al.^12^ reported a significant correlation between the Friedman grade and the volume of palatine tonsils in children who had them surgically removed. Therefore, we used Friedman’s classification for the size of the palatine tonsils. The palatine tonsil size was graded as follows: grade 1, the palatine tonsils were hidden in the pillars; grade 2, the palatine tonsils were located beyond the anterior pillar and between 25% and 50% of the pharyngeal space; grade 3, the palatine tonsils were located beyond the pillars but did not extend the middle and occupied >50% to 75% of the pharyngeal space; and grade 4, the palatine tonsils occupied >75% of the pharyngeal space.

To induce local anesthesia during nasal endoscopy, a cotton pledget soaked with a mixture of 4% lidocaine hydrochloride and 0.05% epinephrine hydrochloride was placed in the nasal cavity, and vasoconstriction was applied 10 minutes before the examination.^13^ Application of a vasoconstrictor is especially necessary in children who frequently present with swelling of the nasal mucous membranes. The whole procedure was performed with the patient in an upright position with their mouth closed, and the outcome was primarily recorded as a video.

Using flexible endoscopy, we graded the pharyngeal tonsils from 1 to 4 based on their relationship to the adjacent structures in the rear of the nasal cavity^14^: grade 1, the pharyngeal tonsils had no contact with other tissues; grade 2, the torus tubarius was in contact with the pharyngeal tonsils; grade 3, the torus tubarius and vomer were in contact with the pharyngeal tonsils; and grade 4, the torus tubarius, vomer, and soft palate were in contact with the pharyngeal tonsils.

The pharyngeal tonsils on both sides of the nasal cavity were graded, and there was no difference between the right and left pharyngeal tonsils in most patients. In patients who showed imbalance between the right and left sides, the higher score was used for analysis.

### Statistical analysis

Continuous data are expressed as mean±standard deviation, and categorical data are expressed as number. The correlation between the palatine and pharyngeal tonsils sizes was analyzed by Spearman’s rank correlation. The associations with the risk of severe OSA were calculated using a multivariable logistic regression model. A p value of <0.05 was considered statistically significant. All statistical analyses were conducted using SPSS for Windows Version 22.0 (IBM Corp., Armonk, NY, USA). The combination of the pharyngeal tonsil grade and palatine tonsil grade was tested for statistical significance with the mean AHI by two-way analysis of variance.

## Results

The mean age, body mass index (BMI), and AHI of the patients were 7.2 years, 16.5 kg/m^2^, and 10.1 per hour, respectively (Table 1). The mean palatine tonsil grade and pharyngeal tonsil grade were 3.0 and 3.0, respectively.

**Figure 1:**
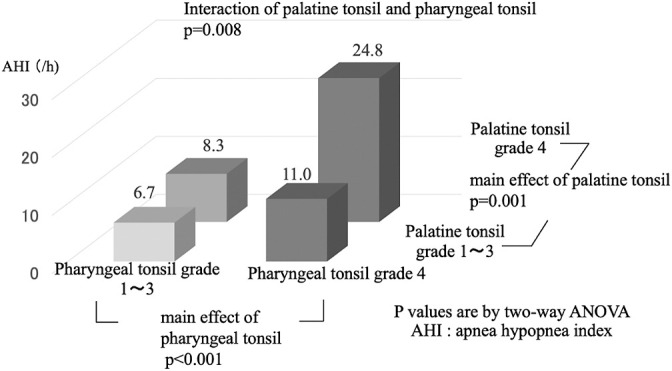
Mean AHI according to palatine tonsil grade and pharyngeal tonsil grade

We divided the palatine and pharyngeal tonsil grades into two groups (grades 1–3 and grade 4) and examined them in combination (Figure 1). Independent of the degree of palatine tonsil hypertrophy, the pharyngeal tonsil grade 4 group had a significantly higher AHI than the grade 1–3 group. Similarly, independent of the degree of pharyngeal tonsil hypertrophy, the palatine tonsil grade 4 group had a significantly higher AHI than the grade 1–3 group. We examined the interaction of the two groups of palatine tonsil grades (1–3 and 4) and the two groups of pharyngeal tonsil grades (1–3 and 4) with the AHI. The results showed that when both the pharyngeal and palatine tonsils were grade 4, the AHI was higher than the AHI expected for the pharyngeal and palatine tonsils alone.

**Figure 2:**
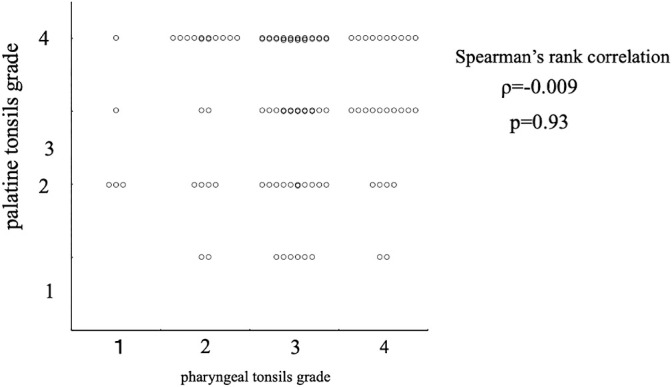
Correlation between pharyngeal tonsils and palatine tonsils

We next examined whether the palatine tonsils and pharyngeal tonsils could be risk factors for severe OSA with an AHI of ≥10 (Table 2). The adjusted odds ratio was 4.42 for palatine tonsil grade 4 versus 1–3 and 10.4 for pharyngeal tonsil grade 4 versus 1–3, and both were highly statistically significant. Table 2 shows the results of the logistic multiple regression analysis of whether the palatine tonsil grade and pharyngeal tonsil grade were possible risk factors for severe OSA with an AHI of ≥10. A logistic regression analysis was performed with severe OSA (AHI of ≥10) as the outcome variable and age, sex, BMI, palatine tonsil grade, and pharyngeal tonsil grade as predictor variables. The patients were divided into two groups according to age: infants (4–6 moths) and children (7–12 years).^15^ The odds ratio for the palatine tonsil grade was 4.42, and the odds ratio for the pharyngeal tonsil grade was 10.40. Although no significant odds ratios were obtained for age or sex, the BMI was involved to a small extent (odds ratio of 1.39). As shown in Figure 2, no significant relationship was found between the palatine tonsil grade and pharyngeal tonsil grade.

## Discussion

One cause of OSA is anatomic narrowing of the upper airway.^16^ Obesity, enlarged tonsils, nasal obstruction, a low soft palate, and enlarged lingual tonsils are the most common causes of anatomic narrowing of the upper airway. In addition to these risk factors, pharyngeal tonsil hypertrophy is an important factor in children.^17–19^

Kang et al.^20^ reported that the combination of pharyngeal tonsil hypertrophy and palatine tonsil hypertrophy increases the risk of developing OSA, which is consistent with our results. In our study, when both the pharyngeal and palatine tonsils were grade 4 and strongly hypertrophied, the mean AHI was more than twice as high. This may be due to the aforementioned double cause of anatomic narrowing of the upper airway, which results in a high degree of upper airway narrowing. Th results of this study suggest that when physical examination reveals hypertrophy of both the pharyngeal and palatine tonsils, the AHI is expected to be high and a definitive diagnosis by PSG should be urgently sought; the possibility of surgical treatment should also be kept in mind.

Kang et al.^20^ and Xu et al.^21^ stated that hypertrophy of the palatine tonsils is associated with a higher risk of developing OSA than is hypertrophy of the pharyngeal tonsils. In our study, the adjusted odds ratio of having severe OSA was 4.42 for the palatine tonsil grade 4 versus 1–3 group and 10.40 for the pharyngeal tonsil grade 4 versus 1–3 group, which may be seen as a difference from previous studies. One reason for this may be the differences in how the pharyngeal tonsils were evaluated, the differences in definition of pharyngeal tonsil hypertrophy, and the fact that other sites of anatomical stenosis (such as nasal obstruction and enlarged lingual tonsils) were not mentioned. We used an endoscope, whereas previous studies evaluated the pharyngeal tonsils using X-ray examination and defined pharyngeal tonsil hypertrophy according to the method described by Fujioka et al.^22^ In cases of grade 4 pharyngeal tonsils, which we defined as pharyngeal tonsil hypertrophy, the nasopharyngeal space is very narrowed. Because the nasopharynx is smaller and less flexible than the mid-pharynx, we speculate that the airway is more likely to be affected when the pharyngeal tonsils occupy the nasopharynx. Therefore, we speculate that the presence of pharyngeal tonsil hypertrophy in our study may have led to a higher risk of OSA than in patients with enlarged palatine tonsils.

This study had three main limitations. First, because it was a single-center study, the number of cases was somewhat small. Second, it did not address other types of anatomic narrowing of the upper airway that can cause OSA, such as nasal obstruction and enlarged lingual tonsils. Finally, the study took place in a specialized hospital focused on sleep-related conditions, and patients were not selected from the local community. Further studies are needed to determine the association between the pharyngeal and palatine tonsil size and sleep disturbances in the healthy population.

## Conclusion

In this study, the pharyngeal tonsils were more intricately involved in severe OSA than were the palatine tonsils. The combination of grade 4 pharyngeal tonsils and grade 4 palatine tonsils resulted in an AHI much higher than the AHI of other combinations (pharyngeal tonsils grades 1–3 and 4, palatine tonsils grades 1–3 and 4). We believe that grade 4 pharyngeal and palatine tonsils have a great influence on severe OSA and that grade 4 pharyngeal tonsils increase the AHI.

## Figures and Tables

**Table1:** Patient’s characteristics of subjects

Number	107
Sex M/F	74/33
Age (year)	7.2±1.8
Height (cm)	123.1±12.3
Weight (kg)	25.6±8.8
Body mass index (kg/m^2^)	16.5±2.7
Pharyngeal tonsil grade	
1	10
2	22
3	28
4	47
Palatine tonsil grade	
1	5
2	20
3	56
4	26

**Table2:** A multivariable logistic regression analysis for severe OSA by palatine tonsils and pharyngeal tonsils grades adjusted for age, gender and body mass index

	OR	95%CI	p value
Age group (year)	0.38	0.13–1.09	0.072
Gender (male)	2.64	0.88–7.91	0.083
Body mass index (kg/m^2^)	1.39	1.12–1.73	0.003
Palatine tonsils grade (4 vs. 1~3)	4.42	1.38–14.17	0.012
Pharyngeal tonsils grade (4 vs. 1~3)	10.40	3.37–32.12	<0.0001

OR=odds ration CI=confidence interval severe OSA=severe obstructive sleep (apnea hypopnea index ≥10) adjusted age group (4~6, 7~14 year), gender (male), body mass index
